# Genetic variation in four maturity genes affects photoperiod insensitivity and PHYA-regulated post-flowering responses of soybean

**DOI:** 10.1186/1471-2229-13-91

**Published:** 2013-06-25

**Authors:** Meilan Xu, Zeheng Xu, Baohui Liu, Fanjiang Kong, Yasutaka Tsubokura, Satoshi Watanabe, Zhengjun Xia, Kyuya Harada, Akira Kanazawa, Testuya Yamada, Jun Abe

**Affiliations:** 1 Research Faculty of Agriculture, Hokkaido University, Sapporo, 060-8589, Japan; 2 National Institute of Agrobiological Sciences, Tsukuba, Ibaraki, 305-8602, Japan; 3 Northeast Institute of Geography and Agroecology, Chinese Academy of Sciences, Harbin, 150040, China

**Keywords:** Photoperiod, Soybean, Flowering, Determinate habit, Post-flowering, Genetic variation

## Abstract

**Background:**

Absence of or low sensitivity to photoperiod is necessary for short-day crops, such as rice and soybean, to adapt to high latitudes. Photoperiod insensitivity in soybeans is controlled by two genetic systems and involves three important maturity genes: *E1*, a repressor for two soybean orthologs of *Arabidopsis FLOWERING LOCUS T* (*GmFT2a* and *GmFT5a*), and *E3* and *E4*, which are phytochrome A genes. To elucidate the diverse mechanisms underlying photoperiod insensitivity in soybean, we assessed the genotypes of four maturity genes (*E1* through *E4*) in early-flowering photoperiod-insensitive cultivars and their association with post-flowering responses.

**Results:**

We found two novel dysfunctional alleles in accessions originally considered to have a dominant *E3* allele according to known DNA markers. The *E3* locus, together with *E1* and *E4*, contained multiple dysfunctional alleles. We identified 15 multi-locus genotypes, which we subdivided into 6 genotypic groups by classifying their alleles by function. Of these, the *e1-as*/*e3*/*E4* genotypic group required an additional novel gene (different from *E1*, *E3*, and *E4*) to condition photoperiod insensitivity. Despite their common pre-flowering photoperiod insensitivity, accessions with different multi-locus genotypes responded differently to the post-flowering photoperiod. Cultivars carrying *E3* or *E4* were sensitive to photoperiod for post-flowering characteristics, such as reproductive period and stem growth after flowering. The phytochrome A–regulated expression of the determinate growth habit gene *Dt1*, an ortholog of *Arabidopsis TERMINAL FLOWER1*, was involved in the persistence of the vegetative activity at the stem apical meristem of flower-induced plants under long-day conditions.

**Conclusions:**

Diverse genetic mechanisms underlie photoperiod insensitivity in soybean. At least three multi-locus genotypes consisting of various allelic combinations at *E1*, *E3*, and *E4* conferred pre-flowering photoperiod insensitivity to soybean cultivars but led to different responses to photoperiod during post-flowering vegetative and reproductive development. The *phyA* genes *E3* and *E4* are major controllers underlying not only pre-flowering but also post-flowering photoperiod responses. The current findings improve our understanding of genetic diversity in pre-flowering photoperiod insensitivity and mechanisms of post-flowering photoperiod responses in soybean.

## Background

Photoperiod sensitivity is an important trait that enables crops to adapt to diverse latitudinal environments. In particular, absence of or low sensitivity to photoperiod is necessary for short-day (SD) crops, such as rice and soybean, to adapt to high latitudes. In soybean (*Glycine max* (L.) Merr.), nine major genes, *E1* through *E8* and *J*, have so far been reported to control time to flowering and maturity (reviewed in [1]). Photoperiod insensitivity is controlled by at least two genetic systems, in which dysfunctional alleles at three maturity loci—*E1*, *E3*, and *E4*—are involved. One control system is ascribed to the double-recessive genotype for *E3* and *E4*[2-6], which encode the phytochrome A (PHYA) proteins GmPHYA3 and GmPHYA2, respectively [7,8]. Together with GmPHYA1, which is encoded by a homoeologous copy of *E4*, these two PHYA proteins redundantly or complementarily function in floral induction and de-etiolation responses under various light conditions [7,8]. *E3* and *E4* direct different flowering responses to long-day (LD) conditions with different red-to-far–red (R:FR) quantum ratios [2-5,9]. *E3* controls the response to light with a high or low R:FR ratio; plants homozygous for the recessive *e3* allele can initiate flowering under the LD conditions generated by fluorescent lamps with a high R:FR ratio [2,9]. *E4* is involved in the response to light with a low R:FR ratio; plants homozygous for the *e3* allele need a recessive *e4* allele to flower under LD generated by incandescent lamps with a low R:FR ratio [3,4,9]. The *e4* allele itself cannot confer the insensitivity to LD conditions induced by both fluorescent and incandescent lamps under the *E3* genetic background [4,5]. The PHYA protein is not only an effective FR sensor but also acts as an R-light photoreceptor under R light with high-photon irradiance [10,11], which is involved, either directly or via interactions with other photoreceptors, in various developmental processes (reviewed in [12]). The *E3* and *E4* genes therefore may participate in a non-additive manner in different aspects of PHYA functions, which are controlled by a single *phyA* gene in *Arabidopsis*. Another *phyA* gene in soybean, *GmphyA1*, has been suggested to function redundantly with *E4* in the de-etiolation response of hypocotyls and floral induction under FR light [7]. Owing to the lack of genetic variants causing phenotypic differences, the function of *GmphyA1* has not yet been determined.

Among the major genes and QTLs that have been reported so far, the *E1* gene has the most prominent effect on flowering time in soybean (reviewed in [1]). Cober et al. [9] used an early-maturing cultivar Harosoy and its near-isogenic lines (NILs) for *E1*, *E3* and *E4* loci to reveal their photoperiod responses to LDs with different R:FR ratios. They found that a NIL with *E1*/*e3*/*e4* was insensitive to R-enriched LD conditions but still retained sensitivity to FR-enriched LD conditions with the low R:FR ratio of 0.9, although the NIL with *e1-as* (originally designed as *e1*, but renamed after [13])/*e3*/*e4* lost the sensitivity across the wide range of R:FR ratios [9]. This result indicates that *E1* has a marked inhibitory effect on flowering, particularly under LD conditions with the low R:FR ratio.

Positional cloning revealed that *E1* encodes a protein that contains a putative bipartite nuclear localization signal and a region related to the B3 domain, a highly conserved domain found in transcription factors in plants [13]. The abundance of *E1* transcripts is under the photoperiodic control regulated by the *E3* and *E4* genes and is negatively correlated with that of two soybean orthologs of *Arabidopsis FLOWERING LOCUS T*, *GmFT2a* and *GmFT5a*[14]. *E1* expression was suppressed under SD conditions regardless of genotype and induced under LD conditions in plants containing either *E3* or *E4* but not in those with the double-recessive *e3e3e4e4* genotype [13]. Furthermore, *E1* over-expression in transgenic T2 soybean plants suppressed the expression of two *GmFT* genes, thereby markedly delaying flowering, suggesting that E1 is a direct repressor of GmFTs [13]. Taken together, the weakened function of PHYA caused by the double-recessive genotype at the *E3* and *E4* loci may ablate or weaken sensitivity to photoperiod, at least partly, through the down-regulation of *E1*.

The other system controlling photoperiod insensitivity in soybean acts through involvement of dysfunctional alleles at the *E1* locus itself. Photoperiod insensitivity in the Japanese landrace ‘Sakamotowase’ has been suggested to be controlled by a different genetic mechanism from PHYA dysfunction, because this landrace has a dominant functional allele at the *E4* locus [6]. Quantitative trait locus (QTL) mapping using testcrosses with a photoperiod-sensitive Harosoy NIL whose genotype was *e1-as*/*e3*/*E4* indicated that the photoperiod insensitivity in Sakamotowase was controlled mainly by an allele at or a gene tightly linked to the *E1* locus, although a minor QTL was detected in linkage group L (Glyma19) [15]. Analysis of the *E1* sequence of Sakamotowase revealed that it contained a dysfunctional allele, *e1-fs*, that produced a loss-of-function truncated protein due to a premature stop codon, which arose as a consequence of a single-base deletion [13]. In contrast, the recessive allele *e1-as* possessed a nonsynonymous substitution in the putative nuclear localization signal, leading to reduced localization specificity of the E1 protein in nucleus and thereby reducing the ability of E1 to suppress expression of the *GmFT* genes [13]. These findings strongly suggest that the molecular basis of the photoperiod insensitivity of Sakamotowase is due, at least in part, to a complete lack of E1 function caused by a dysfunctional allele at the *E1* locus itself. The dysfunctional allele *e1-fs* therefore may provide, singly or together with other unknown genes, another mechanism in the control of photoperiod insensitivity under the presence of *E4* in soybean.

Photoperiod responses of soybean involve not only pre-flowering growth, such as time to flowering, but also post-flowering vegetative and reproductive growth, such as duration of pod filling, development of the terminal inflorescence, and leaf senescence; LD conditions increase reproductive periods and delay leaf senescence and seed maturation of photoperiod-sensitive cultivars [16-18]. Han et al. [16] revealed that the post-flowering growth of a photoperiod-sensitive cultivar was influenced by exposure to R light and reversed by exposure to FR light during the dark period, suggesting that phytochromes control the photoperiod responses of post-flowering growth. However, understanding of the genetic and physiologic bases of post-flowering photoperiod sensitivity is still far from comprehensive.

Soybean maturity genes control not only the time of flowering but also the time to maturation [19-21]. Several QTLs in various linkage groups have been identified to control features of the reproductive period, such as time of maturity and duration of pod filling [22-26]. However, only a few QTLs for post-flowering photoperiod responses have been identified. Cheng et al. [25] found two QTLs that controlled the duration of the post-flowering period under LD conditions. These two QTLs, which appear to correspond to the *E3* and *E8*[27] genes, also were involved in the control of time to flowering [25].

To survey the diverse mechanisms underlying photoperiod insensitivity in soybean and to search for novel genetic factors involved in this process, we first used allele-specific DNA markers to catalog the genotypes of four maturity genes (*E1* through *E4*) in photoperiod-insensitive cultivars and breeding lines introduced from various geographic regions. We also tested the association of these various genotypes with pre- and post-flowering photoperiod responses. Here we report that soybean has gained the trait of photoperiod insensitivity independently and repeatedly through diverse mechanisms. In addition, *E3* and *E4*, together with *E1*, play important roles in not only floral initiation but also post-flowering photoperiod responses, such as maturation and stem termination, in soybean.

## Results

### Effects of photoperiod insensitivity on flowering

As in our previous studies [6,15], we evaluated photoperiod sensitivity according to the difference in the time to flowering (stage R1 [28]) between artificially induced LD and natural daylength (ND) conditions. Harosoy is a photoperiod-sensitive, early-maturing indeterminate cultivar: it possesses a maturity genotype of *e1-as*/*e2*/*E3*/*E4*, and a dominant *Dt1* gene [5]. During the 2-year study period, this cultivar flowered at an average of 57 days after sowing (DAS) under ND conditions of Sapporo, Japan (43º06´N, 141º35´E) but did not produce any flower buds during LD until the end of the artificially induced condition (62 to 65 DAS). Similarly, Harosoy NILs for *e3* (H-*e3*) or *e4* (H-*e4*) did not form any flower buds until the end of the artificially induced LD condition, and they flowered slightly earlier during ND conditions than did Harosoy; the average flowering time was 46 DAS for H-*e3* and 53 DAS for H-*e4*. In contrast, the Harosoy NIL for both *e3* and *e4* (H-*e3*/*e4*) flowered at 40 DAS under both ND and LD, and this line produced pods of 2 cm or longer (stages R4 to R5 [28]) by the end of the artificially induced LD condition. All 53 photoperiod-insensitive accessions used in the current study flowered under LD conditions within 5 days after the date that they flowered under ND conditions.

### Classification of genotypes by use of allele-specific DNA markers

The molecular bases of four maturity genes (*E1* through *E4*) have been determined [7,8,13,29]. To determine the allelic constitutions at the four loci, we genotyped the 4 genes in each of the 53 photoperiod-insensitive accessions by using previously reported allele-specific DNA markers [7,13,29-31].

Four alleles have been identified at the *E1* locus [13]. These alleles include two null alleles: one lacking a 130-kb region harboring the entire gene (*e1-nl*) and the other having a single-base deletion that leads to a frameshift mutation that generates a premature stop codon (*e1-fs*). The conventional recessive allele *e1* (here designated as *e1-as* according to [13]) differs from the dominant allele (*E1*) by a single amino acid substitution in the putative nuclear localization signal; this substitution abolishes nuclear localization of the protein. Eight (seven Japanese and one Chinese) of the 53 accessions had the *E1* allele, 33 had *e1-as*, and 11 had *e1-nl*; only the Japanese landrace ‘Sakamotowase’ had the *e1-fs* allele (Additional file 1).

Two alleles of the *E2* gene, a soybean ortholog of *Arabidopsis GIGANTEA* (*GI*), have been identified: a functional dominant allele (*E2*) and a recessive null allele (*e2*) [29]. All of the 53 accessions we tested had the *e2* allele (Additional file 1).

Two and six alleles have previously been identified at the *E3* and *E4* loci, including one (*e3*) and five (*e4-SORE*-*1*, *e4-kam*, *e4-oto*, *e4-tsu*, and *e4-kes*) null alleles of the *E3* and *E4* loci, respectively [7,8,31]. Of the 53 accessions, 20 had the *E3* allele, and the remaining 33 accessions had the *e3* allele. Fifteen accessions had *E4*, and the remaining 38 accessions had one of four dysfunctional alleles: *e4*-*SORE1* (20), *e4-kam* (2), *e4-oto* (1), and *e4-kes* (15) (Additional file 1). Only 22 accessions were double-recessive homozygotes for the *E3* and *E4* loci, whereas 27 accessions had either of the two dominant alleles, and 4 had both.

### Sequencing of the *E1*, *E3*, and *E4* genes

**Figure 1:**
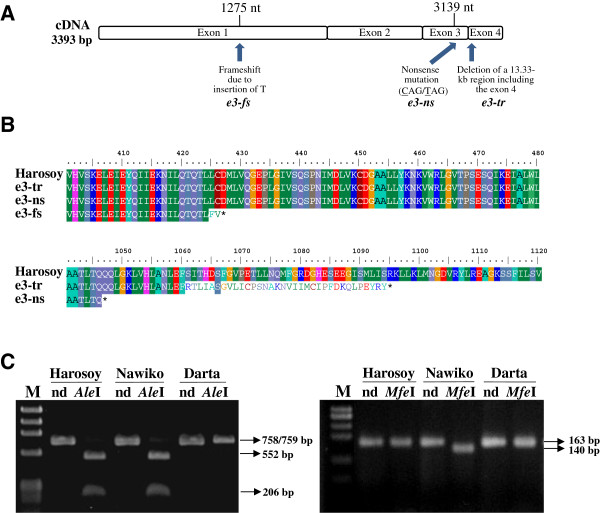
**Novel null alleles detected at the *E3 *locus in photoperiod-insensitive soybean accessions. A**) Positions and types of mutations. **B**) Changes in predicted amino acid sequences; asterisks indicate stop codons. **C**) DNA markers distinguishing each of the recessive alleles from the others. Cultivars Darta and Nawiko have *e3-fs* and *e3-ns*, respectively. Harosoy has a dominant *E3* allele. Marker diagnostic for *e3-fs* shown in left panel. Marker diagnostic for *e3-ns* shown in right panel. nd, not digested.

Genotyping of the four maturity genes by using allele-specific markers indicated that, despite their photoperiod insensitivity, 31 accessions had dominant alleles at either or both of the *E3* and *E4* loci. This result suggests that a novel allele or gene may be involved in the control of this trait. We then sequenced the accessions having at least one dominant *E1*, *E3*, or *E4* allele to examine whether these alleles were truly functional. No sequence variation was detected in the coding region of *E1* in the eight accessions of the *E1*/*e3*/*e4* genotype; all showed the same sequence as the published dominant *E1* allele [13]. Sequence analysis of the *E4* gene similarly revealed no deviations from the published sequence [7] in the 15 accessions tested. However, we found two novel dysfunctional alleles for the *E3* gene among the 18 accessions initially scored as having the *e1*/*E3*/*E4* or *e1*/*E3*/*e4* genotype (Figure 1A and 1B). One of these novel alleles was a nonsense mutation, in which a single-nucleotide substitution from C to T at position 3139 from the adenine of the first codon in exon 3 created a stop codon in place of a codon encoding glutamine. The other novel allele had a premature stop codon in exon 1 that was generated by frameshifting due to the insertion of T at position 1275 in exon 1. We designated these dysfunctional alleles *e3-ns* and *e3*-*fs*, respectively, and renamed the conventional *e3* allele as *e3-tr* (truncated), because it lacks the 3′ region of the gene, including exon 4 [8].

We then developed allele-specific cleaved amplified polymorphic sequence (CAPS) or derived CAPS (dCAPS) markers to reliably identify these dysfunctional alleles (Figure 1C). The *e3-fs* allele can be distinguished from the others after treatment with *Ale*I, which cleaves the 758- or 759-bp amplified product flanking the mutation site into fragments of 552 and 206 bp for the *E3*, *e3-ns*, and *e3-tr* alleles but not *e3-fs* (Figure 1C). The *e3-ns* allele can be distinguished from the others after treatment with *Mfe*I, which uniquely digests the 163-bp amplified *e3-ns* product into fragments of 140 and 23 bp. Most of the accessions, which were originally thought to contain the *E3* allele, had dysfunctional alleles and thus produced truncated GmPHYA3 proteins.

We classified the 53 photoperiod-insensitive accessions into 15 multi-locus genotypes, and then grouped the alleles by function to subcategorize the accessions into 6 genotypic groups (*e1*/*e3*/*e4*, *e1*/*E3*/*e4*, *e1*/*e3*/*E4*, *e1-as*/*e3*/*e4*, *e1-as*/*e3*/*E4*, and *E1*/*e3*/*e4*), where *e1*, *e3*, and *e4* refer to loss-of-function alleles at the respective locus (Table 1). Of these, a combination of dysfunctional alleles at both the *E3* and *E4* loci was predominant: 36 of the 53 accessions shared this genotype. Of the 17 accessions with either the *E3* or *E4* allele, 10 had *e1-fs* or *e1-nl* at the *E1* locus. The remaining seven accessions had the allelic combination of *e1-as*/*e3*/*E4*, the same genotype as that of H-*e3*, which is sensitive to the long days generated by an FR-enriched light source [9,15], such as the incandescent lamps we used in the current study.

**Table 1:** Multi-locus genotype at four maturity loci for 53 early-maturing photoperiod-insenstive soybean accessions

**Multi-locus genotype**^**1)**^	**Allelic combinations at four maturity loci**				**No. of accessions**
	***El***	***E2***	***E3***	***E4***	
*el/e3/e4*	*el-nl*	*e2*	*e3-tr*	*e4-SORE-1*	**2**
*el-as/e3/e4*	*el-as*	*e2*	*e3-ns*	*e4-SORE-1*	**1**
	*el-as*	*e2*	*e3-tr*	*e4-SORE-1*	**8**
	*el-as*	*e2*	*e3-tr*	*e4-kes*	**4**
	*el-as*	*e2*	*e3-fs*	*e4-SORE-1*	**3**
	*el-as*	*e2*	*e3-fs*	*e4-kes*	**10**
*El/e3/e4*	*El*	*e2*	*e3-tr*	*e4-SORE-1*	**4**
	*El*	*e2*	*e3-tr*	*e4-oto*	**1**
	*El*	*e2*	*e3-tr*	*e4-kes*	**1**
	*El*	*e2*	*e3-tr*	*e4-kam*	**2**
*el/E3/e4*	*el-nl*	*e2*	*E3*	*e4-SORE-1*	**2**
*e1/e3/E4*	*el-nl*	*e2*	*e3-tr*	*E4*	**7**
	*el-fs*	*e2*	*e3-tr*	*E4*	**1**
*el-as/e3/E4*	*el-as*	*e2*	*e3-tr*	*E4*	**3**
	*el-as*	*e2*	*e3-fs*	*E4*	**4**

1) lower-case letters (*el*,*e3* and *e4*) indicate loss-of-function alleles at each locus, collectively.

Collectively, all of the 53 photoperiod-insensitive accessions analyzed had a recessive allele at either the *E3* or *E4* locus. When one of these loci had a dominant allele, the *E1* locus always had a loss-of-function *e1*-*fs* or *e1*-*nl* allele or the hypomorphic *e1*-*as* allele.

### Post-flowering photoperiod sensitivity associated with *E3* and *E4*

**Figure 2:**
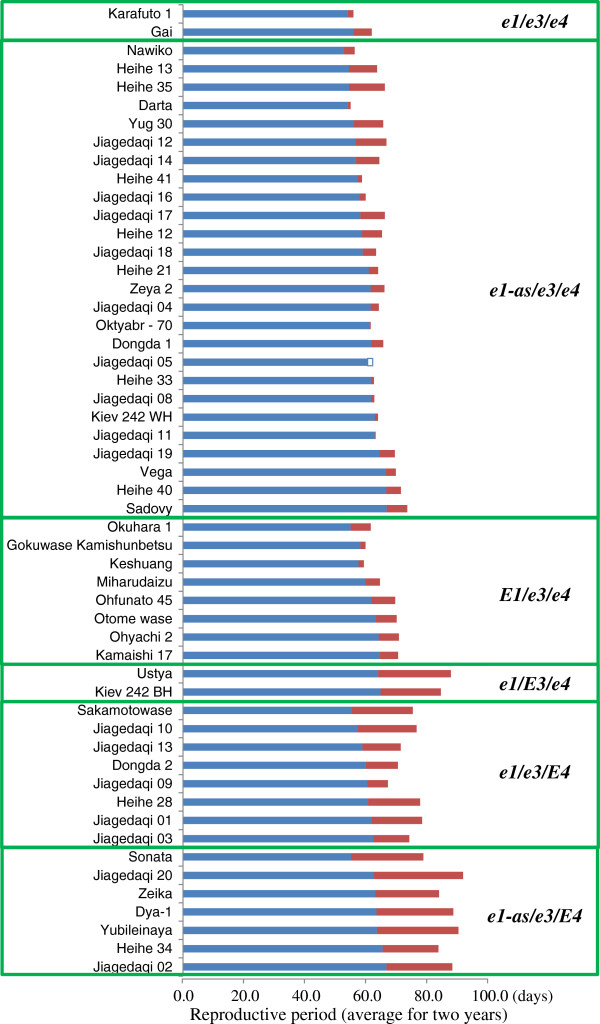
**Variation in reproductive period among photoperiod-insensitive soybean accessions.** Bars indicate the reproductive period (number of days from R1 to R8) under natural-daylength conditions with a maximum of 16.5 h in Sapporo, Japan (blue) and the increase (red) or decrease (open) in duration under artificially induced 20-hour long-daylength conditions. The designations *e1*, *e3*, and *e4* refer to all of the dysfunctional alleles at these respective loci.

**Figure 3:**
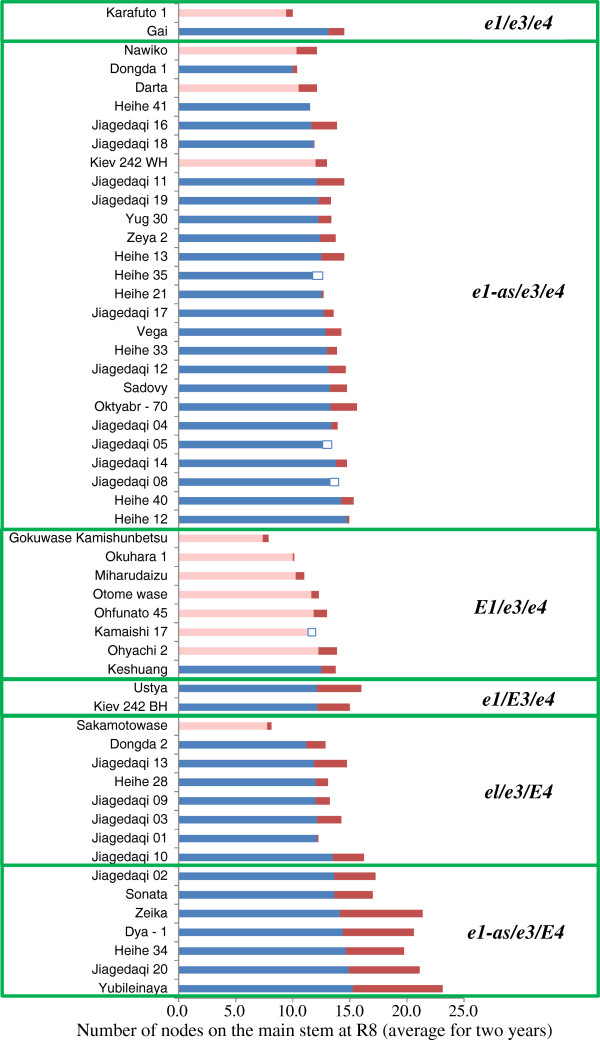
**Variation in the number of nodes on the main stem among photoperiod-insensitive soybean accessions.** Bars indicate the number of nodes produced under natural-daylength conditions with a maximum of 16.5 h in Sapporo, Japan (blue) and the increase (red) or decrease (open) in number of nodes under artificially induced 20-hour long-daylength conditions. Pink indicates the number of nodes produced by determinate *dt1* accessions under natural-daylength conditions. The designations *e1*, *e3*, and *e4* refer to all of the dysfunctional alleles at these respective loci.

The 53 accessions we tested were considered to be photoperiod insensitive in terms of time to flowering, because the difference in flowering dates between ND and LD conditions was small (that is, 5 days or less). However, these accessions differed markedly in their post-flowering vegetative and reproductive growth characteristics, such as reproductive period and stem growth after flowering (Figures 2 and 3). Two-way analysis of variance, in which a combined mean square for interactions by years was used as an error mean square, revealed highly significant (*P* < 0.001) differences for both traits among accessions, daylength conditions, and their interaction.

**Figure 4:**
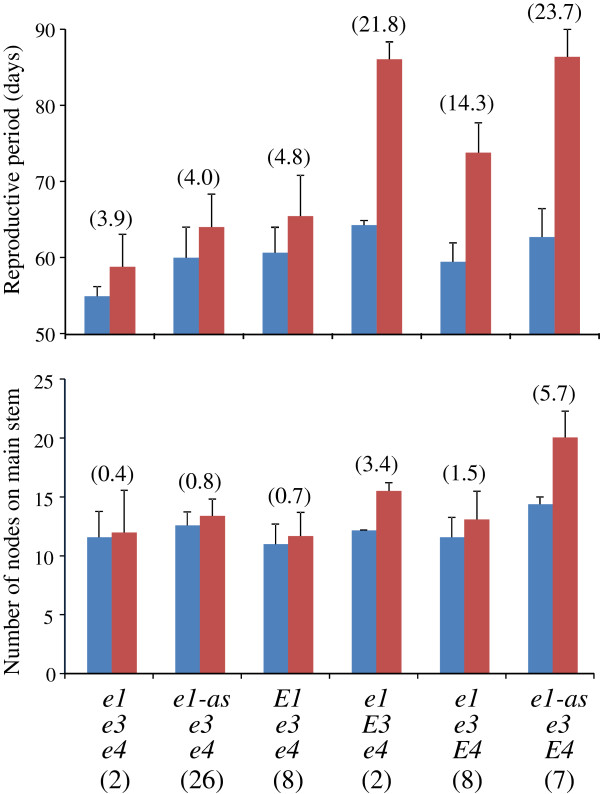
**Average and 1 standard deviation (vertical bars) of reproductive period and number of nodes on main stem under ND (blue bars) and LD (red bars) in photoperiod-insensitive soybean accessions of different maturity genotypes.** The difference between ND and LD is given in parentheses. The genotypic class *E1*/*e3*/*e4* mostly consisted of determinate *dt1* accessions. The designations *e1*, *e3*, and *e4* refer to all of the dysfunctional alleles at these respective loci. Numerals within parentheses under genotypes indicate the number of accessions classified into each multi-locus genotype.

The date of maturation (stage R8 [28]) was delayed more than 5 days during LD in 36 of the 53 accessions tested. This delay was accompanied by slower development of pods or by extension of the flowering period (or both) due to the persistent vegetative activity of apical meristems at the main stem and branches during LD. The difference in reproductive period between ND and LD conditions, evaluated as the number of days from R1 to R8, ranged from -2 to 29 days and varied with the multi-locus genotypes of the accessions (Figure 2; indicated by blue bars with small red bars [increase under LD] or open bars [decrease under LD]). Among the accessions of the *e3*/*e4* genotype, the delay of maturation during LD was a maximum of 12 days, with an average of 4.2 days. In contrast, the reproductive period was elongated by 12 days or more during LD compared with ND in 14 of the 17 accessions having either the *E3* or *E4* allele, with an average of 19.1 days. The difference in reproductive period between ND and LD was, on average across accessions and years, 21.8, 14.3, and 23.7 days in the genotypic groups of *e1*(*e1-nl*)/*E3*/*e4*, *e1*(*e1-nl*/*e1-fs*)/*e3*/*E4*, and *e1-as*/*e3*/*E4*, respectively (Figure 4). This result suggests that *E3* and *E4* were involved in photoperiodic control not only of time to flowering but also of time to pod maturation.

Daylength also influenced stem termination after flowering in soybean (Figure 3). Stem termination in soybean is known to be controlled by at least two genes, *Dt1* and *Dt2*, of which the former has a much greater effect [32]. The *Dt1* gene is an ortholog of *Arabidopsis TERMINAL FLOWER1* (*TFL1*), *GmTFL1b*[33,34]. Four dysfunctional alleles (*dt1-ab*, *dt1-bb*, *dt1-tb*, and *dt1-ta*) have been identified at this locus; all of these mutant alleles produce proteins containing single amino acid substitutions [34]. Genotyping using allele-specific markers revealed that, of the 53 accessions, 41 had the *Dt1* allele, and 12 had the *dt1-bb* or *dt1-tb* allele (Additional file 1).

Determinate cultivars typically terminate stem growth shortly after flowering, whereas indeterminate ones continue stem growth until vegetative growth of the shoot apical meristem (SAM) ends [33]. As expected, our 12 determinate accessions that had the *dt1-bb* or *dt1-tb* allele produced almost the same number of nodes on the main stem during ND and LD (Figure 3; indicated by pink bars with small red or open bar). In contrast, the 41 indeterminate *Dt1* accessions produced more nodes during LD than during ND, and the differences in node number on the main stem between ND and LD varied with the multi-locus genotype (Figure 3; indicated by blue bars with small red or open bar). The difference was less than 2.4 (average, 0.8) nodes among indeterminate accessions with the *e3*/*e4* genotype and was independent of the *E1* genotype (Figure 3); that is, stem growth terminated shortly after flowering under both ND and LD conditions. In contrast, accessions with the *E4* allele tended to produce more nodes during LD than during ND, and this response appeared to be dependent on the genotype (*e1* or *e1-as*) at the *E1* locus (Figures 3 and 4). Accessions having the *e1* (*e1-nl*) allele (*e1*/*e3*/*E4*) terminated stem growth similarly between ND and LD; the difference in node number between ND and LD was, on average, 1.5 nodes across accessions and years (Figure 4). In contrast, accessions having the *e1-as* allele (*e1-as*/*e3*/*E4*) produced at least 3.4 (average, 5.7) more nodes during LD than during ND. Therefore, post-flowering photoperiod growth in soybean, assessed as maturation and stem termination, varied among the accessions tested and with the maturity genotype.

### Control of post-flowering stem termination and pod development by *E3* and *E4*

**Figure 5:**
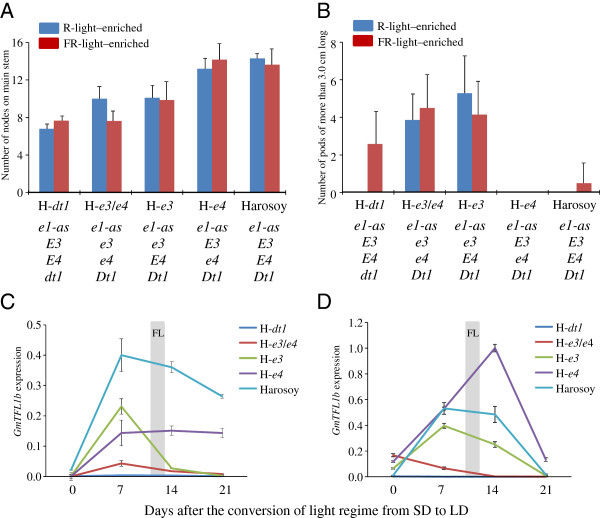
**Different post-flowering photoperiod responses among Harosoy and its NILs for a determinate growth habit allele (*dt1*) and the maturity alleles *e3 *and *e4 *in two independent experiments using different light sources, R-light–enriched [blue] and FR-light–enriched [red] conditions.** In the R-light–enriched condition, a combination of fluorescent and incandescent lamps with an R:FR ratio of 1.2 was used as the light source for 16 hours after dawn followed by lighting with the same light source for an additional 4 hours. The FR-light–enriched condition involved the use of both fluorescent and incandescent lamps with an R:FR ratio of 1.2 for 16 hours after dawn followed by lighting with incandescent lamps only for an additional 4 hours. **A**) Number of nodes (average ± 1 standard deviation) on the main stem at 30 days after flowering. **B**) Number of pods (average ± 1 standard deviation) more than 3.0 cm long at 30 days after flowering. Transcripts levels of *Dt1* (*GmTFL1b*) at stem tips in different growth stages under (**C**) R-light–enriched and (**D**) FR-light–enriched conditions. Relative transcript levels (mean and standard error; *n* = 3) were analyzed by quantitative RT-PCR and normalized to *β-tubulin* (*TUB*). FL indicates time of flowering.

Han et al. [16] revealed that the post-flowering growth of a photoperiod-sensitive cultivar is influenced by the photoreaction that can be induced by exposure to R light and reversed by subsequent FR light exposure during the dark period. This pattern implies that phytochromes are involved in the photoperiod responses for post-flowering development in soybean. To confirm the different responses of post-flowering growth to photoperiod that we observed among the maturity genotypes in the field experiment, we analyzed pod development, stem termination, and the expression pattern of *Dt1* in stem tips under LD for Harosoy (*E3*/*E4*/*Dt1*) and its NILs for *e3*, *e4*, and *dt1*. We induced flowering in these lines as described in the Methods section; SD treatment until 12 days after emergence (DAE) is sufficient to initiate and maintain flowering of Harosoy under non-inductive LD conditions [14]. The soybean maturity genes, *E3* and *E4*, have been characterized in detail for the responses to long days with different R:FR ratios [5,9]. We thus used two LD conditions with different light sources, R-light-enriched and FR-light-enriched, to discriminate the functions of *E3* and *E4*. Harosoy and its NILs for *e3*, *e4*, and *dt1* (*dt1-bb*) flowered at almost the same time (11 to 13 days after the conversion to LD conditions; Figure 5C, D) under two lighting conditions. Stem growth terminated at the 6th to 8th node in the Harosoy NIL for *dt1* (H-*dt1*); indeterminate lines produced more nodes than did H-*dt1*, and the number of nodes produced until the end of experiment (30 days after flowering) varied with the maturity genotype (Figure 5A). Compared with H-*dt1*, Harosoy and H-*e4* produced, on average, 7.5 and 6.4 more nodes under the R-light–enriched condition and 5.9 to 6.5 nodes more under the FR-light–enriched condition, respectively. H-*e3*/*e4* and H-*e3* terminated stem growth earlier than did Harosoy and H-*e4* under both conditions. In addition, stem growth terminated earlier in H-*e3*/*e4* than in H-*e3* under the FR-light–enriched condition, and H-*e3*/*e4* produced almost the same number of nodes as did H-*dt1*.

Pod development showed a pattern slightly different from the results observed for stem growth (Figure 5B). Under the R-light–enriched condition, Harosoy, H-*e4*, and H-*dt1* did not produce any pods longer than 3.0 cm until 30 days after flowering, although they flowered at almost the same time as did H-*e3* and H-*e3*/*e4*. In contrast, H-*e3* and H-*e3*/*e4* produced, on average, 5.3 and 3.9 3-cm or longer pods under the R-light–enriched condition. In contrast, under the FR-light–enriched condition, H-*e3* and H-*e3*/*e4* produced 4.1 and 4.5 pods, respectively, whereas Harosoy yielded only a few pods (0.5), and H-*e4* made no pods; H-*dt1* produced an average of 2.6 pods (Figure 5B). The pod development of H-*dt1* in the FR-light–enriched condition may indicate that an R-light–enriched condition has a larger effect in controlling the development of pods after flowering than does the FR-light–enriched condition.

*Dt1* expression in determinate soybean plants rapidly decreases concomitant with floral induction, whereas this expression is maintained for a while after flowering starts in indeterminate accessions [33]. Here we found that the expression of *Dt1* in the stem tips of 12-day-old plants grown in SD was very low under both R- and FR-enriched conditions, regardless of the genotype at the *E3* and *E4* loci (Figure 5C and 5D; see data regarding 0 day). In addition, the expression level in H-*dt1* remained very low at later growth stages. In contrast, the indeterminate lines (except H-*e3*/*e4*) exhibited rapid increases in *Dt1* expression at 7 days after conversion to LD (19-day-old plants; 7 days in Figure 5C and 5D). Thereafter, Harosoy and H-*e4* maintained *Dt1* expression at relatively high levels until 21 days after conversion to LD under the R-light–enriched condition (Figure 5C), but *Dt1* expression decreased rapidly at 14 days and afterward under the FR-light–enriched condition (Figure 5D). H-*e3* had a similar but slightly lower level of expression than did Harosoy and H-*e4* under the FR-light–enriched condition (Figure 5D), but the abundance of *Dt1* transcripts decreased rapidly at 14 days after conversion to LD in the R-light–enriched condition (Figure 5C). In contrast to that in Harosoy, H-*e3*, and H-*e4*, *Dt1* expression was low (similar to that in H-*dt1*) in H-*e3*/*e4* throughout all growth stages. These results therefore suggest that *Dt1* expression is under the control of two *phyA* genes, *E3* and *E4*, for control of post-flowering growth, although a direct causative relationship between *Dt1* expression and the two *phyA* genes should be addressed in a further study. This notion is in accordance with PHYA-regulated signal transduction of *Dt1* expression, which was expected given the presence in the *Dt1* promoter region of a sequence identical to the SORLIP1 (sequence overrepresented in light-induced promoter 1 *cis*-element [33]). The *cis*-element sequence found in *Dt1* is the most common *cis*-element in SORLIPs in *Arabidopsis* genes that are induced or repressed by FR light [35].

## Discussion

### Three major genetic groups confer photoperiod insensitivity in soybean

In the current study, we assessed the genotypes of 53 photoperiod-insensitive soybean cultivars at four maturity genes and a determinate growth-habit gene by using allele-specific DNA markers that distinguished each of the recessive alleles from the others. These accessions have been introduced from various high-latitude geographic regions. Our first goal was to determine whether there was any association between the genotypes at four major maturity loci (*E1* through *E4*) and photoperiod sensitivity. Our second goal was to reveal any novel genetic factors that controlled photoperiod insensitivity in these diverse genetic resources. The photoperiod-insensitive cultivars and breeding lines we tested were classified into three genotypic groups according to the functions of the alleles at the respective loci: the *e3*/*e4* group; the group containing *e1* (*e1*-*nl* or *e1-fs*) and either *E3* or *E4*; and the *e1-as*/*e3*/*E4* group.

All of the 53 photoperiod-insensitive accessions tested had the dysfunctional allele at the *E2* locus. The *E2* gene is a soybean ortholog of *Arabidopsis GIGANTEA* (*GI*) [29]. In *Arabidopsis thaliana*, *GI* regulates *FT* expression through multiple mechanisms: 1) GI binds to FLAVIN-BINDING, KELCH REPEAT, F BOX protein 1, leading to degradation of a key *CO* repressor (CYCLING DOF FACTOR 1), upregulation of the expression of *CONSTANS* (*CO*), and subsequent activation of *FT* expression [36,37]; 2) GI regulates the expression levels of miRNA172, whose targets encode repressors of *FT*, such as TARGET OF EAT 1 and SCHLAFMUTZE [38,39]; and 3) GI modulates the stability or promoter accessibility of various *FT* repressors [40], including SHORT VEGETATIVE PHASE [41], TEMPRANILLO (TEM) 1, and TEM2 [42] Accordingly, the dysfunctional allele at the *E2* locus likely is required for photoperiod insensitivity in these soybean accessions.

One of the three genotypic groups consisted of accessions that possessed the double-recessive genotype (*e3*/*e4*) at the *E3* and *E4* loci. These accessions accounted for approximately 70% (36 of 53) of the photoperiod-insensitive accessions tested, suggesting that dysfunction of PHYA is the most common mechanism underlying photoperiod insensitivity in soybean.

Another genotypic group comprised the accessions having the dysfunctional *e1* alleles (*e1-nl* or *e1-fs*) in combination with either a dominant *E3* or *E4* allele. This group contained Sakamotowase, which has the *e1-fs* allele that yields a nonfunctional E1 protein, as has been revealed through transient expression assays [13]. In addition, the major QTL for photoperiod insensitivity in this line is tightly linked to the *E1* locus [15]. These observations suggest that the photoperiod insensitivity of Sakamotowase can be ascribed to the complete lack of E1 function. Another dysfunctional allele, *e1-nl*, which lacks the entire *E1* gene [13], occurred fairly frequently (11 of 53 lines) among the photoperiod-insensitive accessions we tested. The dysfunctional alleles *e1-fs* and *e1-nl* may have similar roles in abolishing or weakening the photoperiod responses regulated by the *E3* or *E4* allele. Additional research is needed to determine whether these dysfunctional alleles at the *E1* locus can singly lead to photoperiod insensitivity in the presence of the *E3* or *E4* allele.

The remaining genotypic group consisted of the accessions with the allelic combination of *e1-as*/*e3*/*E4*. The Harosoy NIL for *e3* also has the same allelic combination. However, this NIL is sensitive to the LD conditions generated by using an FR-enriched light source (like that we generated by using incandescent lamps in the current study), although it did not respond to the R-enriched LD condition [4-6,9]. Therefore, the allelic combination of *e1-as*/*e3*/*E4* is not sufficient to confer photoperiod insensitivity. It is conceivable that a novel gene—different from the dysfunctional alleles at the *E1*, *E3*, and *E4* loci—may be involved in the control of the photoperiod insensitivity of accessions of this genotype under the FR-enriched LD condition. *GmphyA1*, a homoeolog of *E4*, is a possible candidate for this photoperiod-insensitivity controller: *GmphyA1* has been suggested to function in a redundant manner to the *E4* allele in the de-etiolation responses of hypocotyls and in flowering under the FR-enriched LD condition [7,9]. The dysfunctional allele at the *E4* locus has adverse effects on plant morphogenesis, such as an impaired de-etiolation response under FR light [7] and the production of longer internodes under LD conditions relative to the *E4* allele [43], making the plant susceptible to lodging. Accordingly, the use of the dominant *E4* allele may contribute to the lodging tolerance of photoperiod-insensitive cultivars if another factor conditions the insensitivity. An appropriate combination of alleles should therefore be selected from among those representing diverse genetic mechanisms to adapt soybean cultivars to the conditions of the target environment.

Multiple dysfunctional alleles have been detected for all of the *E1*, *E3*, and *E4* loci [13,31], including two novel alleles at the *E3* locus [this study]. Our previous studies indicated that five loss-of-function alleles at the *E4* locus originated recently and independently in different soybean landraces from East Asia [31,44]. These results suggest that photoperiod insensitivity in soybean has arisen redundantly through multiple combinations of independently generated alleles at the *E1*, *E3*, and *E4* loci.

### Two *phyA* genes, *E3* and *E4*, control post-flowering responses in soybean

Another finding of the current study is that vegetative and reproductive growth characteristics after flowering, such as maturation and stem termination, were influenced by photoperiod responses regulated by the *E3* and *E4* loci. Our findings are therefore in good agreement with previous observations regarding an association between photoperiod sensing and post-flowering responses [16-18] and involvement of phytochrome(s) in post-flowering responses [16]. We found that soybean accessions with different photoperiod-insensitive multi-locus genotypes responded differently to the photoperiod after flowering. Despite their photoperiod insensitivity in regard to time to flowering, accessions that had either the *E3* or *E4* allele matured at least 10 days later under LD than ND conditions (Figures 2 and 4). Furthermore, vegetative activity of the SAM during LD persisted through late growth stages in the indeterminate accessions with allelic combinations of *e1*/*E3* or *e1*-*as*/*E4* to produce more nodes than did those grown under ND condition (Figures 3 and 4). These behaviors are in sharp contrast to those of the accessions of the *e3*/*e4* group, which, independent of the type of allele or genotype at the *E1* locus, matured without notable delays during LD relative to ND and terminated stem growth similarly under these two conditions (Figure 4).

The different photoperiod responses of post-flowering vegetative and reproductive growth that we observed among the various maturity genotypes were further confirmed through analyses with Harosoy and its NILs for *e3*, *e4*, and *dt1*. Plants among the NILs in which we induced flowering by SD treatment for 12 DAE exhibited different stem and pod growth under subsequent LD conditions. In particular, the NILs with the *E3* allele retained the vegetative activity of the SAM of flower-induced plants and inhibited the pod development after flowering during LD conditions, whereas those with a recessive *e3* allele terminated stem growth shortly after flowering to produce fewer nodes and developed pods under both of the light regimens (Figures 5A and 5B). We also detected a slight effect of *e4* only in the genetic background of *e3* under the light regimen in which only FR-enriched lights were used to provide four hours of additional daytime subsequent to the 16-hour daytime period (Figure 5A). Our present results strongly suggest that post-flowering photoperiod responses in soybean are controlled by the *E3* and *E4* genes.

### A proposed gene regulatory network for pre- and post-flowering photoperiod responses in soybean

**Figure 6:**
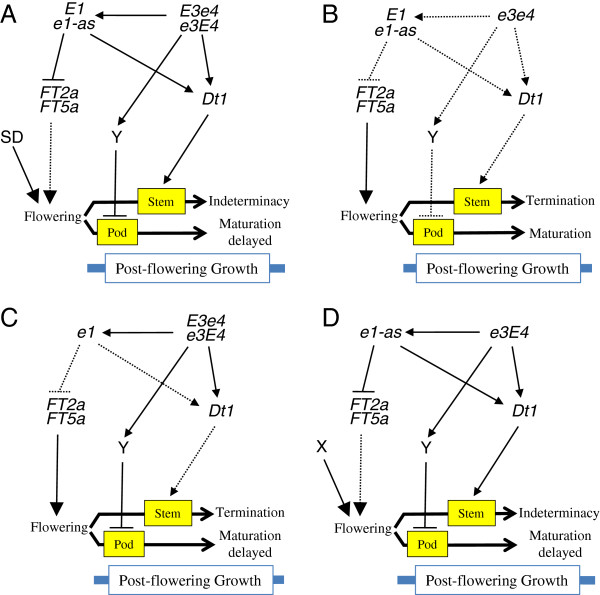
**Proposed model of the genetic network comprising three maturity genes and one growth-habit gene affecting pre- and post-flowering photoperiod responses in soybean under LD. A**) Photoperiod-sensitive plants with *E1* or *e1*-*as* and either *E3* or *E4*. PHYA-regulated *E1* expression inhibits the expression of soybean orthologs of *FT* (*GmFT2a* and *GmFT5a*). Once induced by brief exposure to SD, flowering persists after the transfer to LD, but seed maturation and stem termination are inhibited under the control of PHYA encoded by the *E3* and *E4* alleles through activation of an unknown factor (Y) and upregulation of *Dt1* expression, respectively. **B**) Photoperiod-insensitive plants of the *e3*/*e4* group. Owing to the dysfunction of PHYA, flowering is induced under LD, which is followed by normal seed maturation and stem termination. **C** and **D**) Photoperiod-insensitive plants of **C**) the *e1*/*E3*/*e4* or *e1*/*e3*/*E4* group and D) the *e1-as*/*e3*/*E4* group. In the *e1*/*E3*/*e4* or *e1*/*e3*/*E4* group, flowering is induced by the dysfunction of the *E1* gene (panel **C**); whereas that in the *e1-as*/*e3*/*E4* group is induced through an unknown factor (X) (panel **D**). Seed maturation of plants in these two groups is delayed similarly because of the presence of the *E3* or *E4* allele. However, stem growth after LD terminates earlier in the *e1*/*E3*/*e4* or *e1*/*e3*/*E4* group than in the *e1*-*as*/*e3*/*E4* group: PHYA-mediated *Dt1* expression under LD likely preserves the vegetative activity of the SAM to produce more nodes in the *e1*-*as*/*e3*/*E4* group. The designations *e1*, *e3*, and *e4* refer to all dysfunctional alleles at these loci. Solid and dotted arrows indicate activation and lack of activation, respectively.

We have summarized our current results as a gene network involving three maturity genes (*E1*, *E3*, and *E4*), a determinate habit gene (*Dt1*), and two *GmFT*s (*GmFT2a* and *GmFT5a*), that regulates the pre-flowering and post-flowering photoperiod responses of soybean under LD (Figure 6). In photoperiod-sensitive plants having the *E1* or *e1*-*as* allele and a dominant allele at either the *E3* or *E4* locus (or both) (Figure 6A), we propose that flowering is not induced under FR-enriched LD [3-5,9] because the *E1* and *e1*-*as* alleles inhibit the gene expression of *GmFT2a* and *GmFT5a*[13]. When those plants are exposed to a short period of SD, flowering is induced and persists even after the transfer to non-inductive LD, but subsequent seed maturation is delayed through the influence of an as yet unknown factor (Y), and vegetative activity at SAM is retained to produce more nodes due to *Dt1* expression. Both factor Y and *Dt1* are under the control of PHYA encoded at the *E3* or *E4* locus (Figure 6A).

We propose that flowering in photoperiod-insensitive plants of the *e3*/*e4* group, which has dysfunctional alleles at the *E3* and *E4* loci, is induced through the upregulation of *GmFT*s in the absence of *E1* expression [13]. Flowering in *e3*/*e4* plants is followed by normal seed maturation and stem termination, possibly due to downregulation of Y and *Dt1*, respectively (Figure 6B). The delayed maturity in some of the *e3/e4* accessions in LD compared with ND (Figure 2 and 4) may be ascribed to the presence of a copy of the *phyA* gene at a different locus (*GmphyA1*) [7] or other photoreceptors.

In contrast, in photoperiod-insensitive plants that have a functional *phyA* gene at either the *E3* or *E4* locus, we suggest that flowering is induced under LD either by loss-of-function alleles at the *E1* locus in the *e1*/*e3*/*E4*–*e1*/*E3*/*e4* group (Figure 6C) or by an unknown genetic factor (X) in the *e1*-*as*/*e3*/*E4* group (Figure 6D). In contrast to the *e3*/*e4* group, seed maturation in these two groups is delayed similarly under LD due to regulation by a functional *phyA* gene (*E3* or *E4*). However, stem growth under LD differs between these groups: stem growth terminates earlier in the *e1*/*e3*/*E4*–*e1*/*E3*/*e4* group than in the *e1*-*as*/*e3*/*E4* group (Figures 3 and 4). In *e1*-*as*/*e3*/*E4* plants, PHYA-mediated *Dt1* expression under LD may preserve vegetative activity at SAM to produce more nodes, as indicated by the post-flowering responses of Harosoy and its NILs for *e3* or *e4* in LD (Figure 5).

These models prompt two questions regarding post-flowering growth in soybean. First, how does the *E1* gene influence stem growth after flowering? Apart from its effect on flowering and maturity, the role of the *E1* gene in morphogenesis has not been determined, although it indeed influences various morphologic traits, yield, and other traits such as tolerance to chilling temperature [45,46]. The *E1* and *e1*-*as* alleles inhibit the expression of *GmFT2a* and *GmFT5a*, which are under the control of *phyA* genes [13], whereas the loss-of-function alleles *e1*-*fs* and *e1*-*nl* do not. In *Arabidopsis*, FT protein binds to FLOWERING LOCUS D (FD) and promotes the expression of *APETALA1* and *LEAFY*, flower meristem genes [47-49]; reviewed in [50], which, in turn, suppresses *TFL1* transcription and terminates stem growth [51,52]. Therefore, the different effects of the alleles at the *E1* locus on post-flowering stem growth might be ascribed not to a direct effect but rather to an indirect effect through suppression of *GmFT* expression.

Another question is whether flowering itself is a direct trigger for seed development in soybean. The findings we obtained in the current study indicate that PHYA-mediated photoperiod responses may regulate seed maturation directly or indirectly via an as yet unknown factor: only when two *phyA* genes were dysfunctional did seed maturation after flowering under LD proceed without any marked delay (Figure 6). Additional research therefore should explore key factors (such as Y in our model) in promoting seed development. Comparing expression profiles during early seed development between SD-grown plants and those whose reproductive growth has reverted to a vegetative pattern may advance our understanding of the molecular bases of seed development in soybean.

## Conclusions

The present study revealed diverse genetic mechanisms underlying photoperiod insensitivity in soybean. At least three multi-locus genotypes comprising various allelic combinations at the *E1*, *E3*, and *E4* loci can confer pre-flowering photoperiod insensitivity. These genotypes responded differently to photoperiod during post-flowering reproductive development, indicating involvement of the *phyA* genes *E3* and *E4*. Our results further indicate that flowering itself may not necessarily be a direct trigger for seed development. *E1*, *E3*, and *E4* control the photoperiod responses for pre- and post-flowering development, which directly influences final seed yield in soybean.

## Methods

### Plant materials

We used 53 photoperiod-insensitive soybean (*Glycine max*) accessions in the current study (Additional file 1). These included 9 accessions from northern Japan (6 from Hokkaido and 3 from the Tohoku region), 29 from northeast China, 8 from far-Eastern Russia, 4 from Ukraine, and 3 from Poland. Harosoy (PI548573) and its NILs for *e3* (PI547716; H-*e3*), *e4* (PI591435; H-*e4*), both *e3* and *e4* (PI546043*;* H-*e3*/*e4*), and *dt1* (PI547687; H-*dt1*) were used as controls to delineate pre- and post-flowering photoperiod insensitivity.

### Evaluation of photoperiod sensitivity

Photoperiod sensitivity was evaluated based on differences in the date when the first flower opened (stage R1 [27]) between plants grown under artificially induced LD conditions and those under ND conditions. The experiment was performed in an experimental field with outdoor lighting at Hokkaido University, Sapporo, Japan (43°06′N, 141°35′E) during 2011 and 2012. ND conditions in Sapporo, including civil twilight, reached a maximum of 16.5 h. Seeds were sown in paper pots (No. 2, Nippon Tensai Tougyo, Obihiro, Japan) on June 1, 2011, and May 29, 2012, and 10-day-old seedlings were transplanted into both LD and ND fields. LD conditions were generated by using 500-W incandescent lamps placed 2 m above the soil surface at intervals of 4 m. Lights were on from 0300 to 0600 and from 1830 to 2300 until the end of treatment (August 2). Under illumination by incandescent lamps, the R:FR (660:730) quantum ratio was 0.72, and the average photosynthetic photon flux at the canopy surfaces was 1 μmol photon sec^-1^ m^-2^, as measured at night by using a quantum sensor (model LI-1800C, Li-Cor, Lincoln, NE). The plants were checked every other day to determine stages R1 and R8 [27]. The nodes on the main stem were counted at R8.

### Genotyping by using allele-specific DNA markers

We genotyped four maturity genes (*E1* through *E4*) and a growth habit gene (*Dt1*) by using allele-specific DNA markers for *E1*[13], *E2*[29], *E3*[30], *E4*[7,31], and *dt1* (*dt1-bb*) [33]. We used available sequence information [34] to develop DNA markers for *dt1-ab* and *dt1-tb*; we did not develop a DNA marker for *dt1-ta* because of its rarity [34]. Primer sequences, DNA marker type, restriction enzyme used, and resultant fragment sizes for each marker are shown in Additional file 2. Total genomic DNA was extracted from trifoliate leaves as described previously [53]. Each PCR reaction contained 30 ng of total genomic DNA as template and *ExTaq* polymerase (TaKaRa, Otsu, Japan); amplification conditions were 30 cycles at 94°C for 30 sec, 56 to 60°C (depending on the primers used) for 30 sec, and 72°C for 30 to 90 sec. PCR products or those digested with appropriate restriction enzymes as needed were separated by electrophoresis in 1% to 3% (w/v) agarose gels, stained with ethidium bromide, and visualized under UV light.

### Sequence analyses

Accessions having at least one dominant allele of *E1*, *E3*, or *E4* were sequenced. A 525-bp region for *E1*, four regions of 571 to 2350 bp covering each of four exons for *E3*, and two overlapping fragments of 2384 and 3506 bp that covered the entire *GmphyA2* coding sequence for *E4* were amplified from genomic DNA by using KOD FX (Toyobo Life Science, Osaka, Japan). Amplified fragments were purified by using the ExoSAP-IT enzyme kit (GE Life Sciences Japan, Tokyo, Japan). The purified PCR products were used as templates for forward and reverse sequencing reactions generated by using a BigDye Terminator v3.1 Cycle Sequencing kit and ABI PRISM 3100 Avant Genetic Analyzer (Applied Biosystems Japan, Tokyo, Japan) according to the manufacturer’s instructions.

### Development of DNA markers for novel *E3* loss-of-function alleles

We used sequences flanking mutation sites to develop allele-specific CAPS and dCAPS DNA markers. The targeted region for each mutation was amplified from the DNA preparations by using *ExTaq* polymerase with primers specific to each mutation. The PCR products were digested with appropriate restriction enzymes (Additional file 2), separated by electrophoresis in 1% or 2.5% agarose gels, stained with ethidium bromide, and visualized under UV light.

### Expression analysis for the determinate growth habit gene *Dt1*

Time-course–dependent expression of the *Dt1* (*GmTFL1b*) gene, an *Arabidopsis TERMINAL FLOWER1* (*TFL1*) ortholog, at the stem tip was analyzed for Harosoy and its NILs for *dt1*, *e3*, and *e4*. Plants were grown in a growth chamber with a constant air temperature of 25°C, an average photon flux of 300 μmol photons m^-2^ s^-1^, and an R:FR ratio of 1.2 provided by a combination of fluorescent and incandescent lamps. The daylength was set at 12 hours until 12 DAE and at 20 hours thereafter. Two independent experiments were done by using different light sources. Specifically, in experiment 1, a combination of fluorescent and incandescent lamps with an R:FR ratio of 1.2 was used as the light source for 16 hours after dawn followed by lighting with the same light source for an additional 4 hours; experiment 2 involved the use of both fluorescent and incandescent lamps with an R:FR ratio of 1.2 for 16 hours after dawn followed by lighting with incandescent lamps only for an additional 4 hours. In both experiments, stem tips were collected in bulk from four individual plants at 4 zeitgeber time every 7 days beginning at 12 DAE. The samples were immediately frozen in liquid N_2_ and stored at -80°C.

Transcript levels of *Dt1* were determined by quantitative real-time PCR (qRT-PCR). The qRT-PCR mixture was prepared by mixing 1 μL of the cDNA synthesis reaction, 5 μL 1.2 μM primer premix, 10 μL SYBR Premix *ExTaq* Perfect Real Time (TaKaRa, Otsu, Japan), and water to yield a final volume of 20 μL. The analysis was done by using the CFX96 Real-Time System (BIO-RAD Laboratories Japan, Tokyo, Japan). The primers used were 5′-AGGCACAACAGATGCCACAT-3′ and 5′-GGCAAAACCAGCAGCTACTT-3′ for *GmTFL1b* (*Dt1*) and 5′-GAGAAGAGTATCCGGATAGG-3′ and 5′-GAGCTTGAGTGTTCGGAAAC-3′ for *β-tubulin*. The PCR cycling conditions were 95°C for 3 min followed by 40 cycles of 95°C for 10 sec, 58°C for 20 sec, 72°C for 20 sec, and 78°C for 2 sec. Fluorescence was quantified before and after incubation at 78°C to monitor the formation of primer dimers. A reaction mixture without reverse transcriptase was included as a control to confirm that no amplification resulted from genomic DNA contaminants in the RNA sample. In all PCR experiments, amplification of a single DNA species was confirmed by both melting curve analysis of qRT-PCR and gel electrophoresis of PCR products. The mRNA level of *GmTFL1b* was normalized to that of *β-tubulin*.

### Accession numbers

Sequence data from this article can be found in the GenBank/EMBL/DDBJ data libraries under the accession numbers AB766210 (exon 1 of the *e3-fs GmphyA3* allele) and AB766211 (exon 3 of the *e3-ns GmphyA3* allele).

## Abbreviations

QTL: Quantitative trait locus; NIL: Near-isogenic line; PCR: Polymerase chain reaction; qRT-PCR: Quantitative real-time PCR; CAPS: Cleaved amplified polymorphic sequence; dCAPS: Derived CAPS; PHYA: Phytochrome A; FT: FLOWERING LOCUS T; GI: GIGANTEA; CO: CONSTANS; TFL1: TERMINAL FLOWER1; SAM: Shoot apical meristem; DAS: Days after sowing.

## Competing interests

The authors declare that they have no competing interests.

## Supplementary Material

Additional file 1 Genotypes at four maturity loci and a determinate growth habit locus in 53 photoperiod-insensitive soybean accessions of different origins, as estimated by using allele-specific DNA markers. Click here for file

Additional file 2 **Allele-specific DNA markers that distinguish recessive alleles from dominant functional ones at the maturity loci *E1*, *E2*, *E3*, and *E4 *and the determinate growth habit locus *Dt1 *in soybean.** Click here for file
