# Supplementary material for: Genetic variation in four maturity genes affects photoperiod insensitivity and PHYA-regulated post-flowering responses of soybean
Source: BMC Plant Biol. 2013 Jun 25;13:91. doi: 10.1186/1471-2229-13-91 (PMC3698206; doi:10.1186/1471-2229-13-91)
Supplement: Additional file 1 — Genotypes at four maturity loci and a determinate growth habit locus in 53 photoperiod-insensitive soybean accessions of different origins, as estimated by using allele-specific DNA markers. [file 1471-2229-13-91-S1.pdf]

Additional file 1. Genotypes at four maturiy and a determinate growth habit loci for 53 early-matuiring photoperiod-insenstive soybean accessions of different origins

Accession	Origin	Genotype				
		E1	*E2*	*E3*	*E4*	*Dt1*
Harosoy-e3e4		*e1-as*	*e2*	*e3-tr*	*e4-SORE1*	***Dt1***
Harosoy-e3		*e1-as*	*e2*	*e3-tr*	***E4***	***Dt1***
Harosoy-e4		*e1-as*	*e2*	***E3***	*e4-SORE1*	***Dt1***
Harosoy		*e1-as*	*e2*	***E3***	***E4***	***Dt1***
Gokuwase Kamishunbetsu	Hokkaido/Japan	***E1***	*e2*	*e3-tr*	*e4-SORE1*	*dt1-bb*
Kamaishi 17	Tohoku/Japan	***E1***	*e2*	*e3-tr*	*e4-kam*	*dt1-bb*
Keshuang	North-east China	***E1***	*e2*	*e3-tr*	*e4-kes*	***Dt1***
Miharudaizu	Hokkaido/JPN	***E1***	*e2*	*e3-tr*	*e4-SORE1*	*dt1-bb*
Ohfunato 45	Tohoku/Japan	***E1***	*e2*	*e3-tr*	*e4-kam*	*dt1-bb*
Ohyachi 2	Hokkaido/Japan	***E1***	*e2*	*e3-tr*	*e4-SORE1*	*dt1-bb*
Okuhara 1	Hokkaido/Japan	***E1***	*e2*	*e3-tr*	*e4-SORE1*	*dt1-bb*
Otome wase	Tohoku/Japan	***E1***	*e2*	*e3-tr*	*e4-oto*	*dt1-bb*
Darta	Poland	*e1-as*	*e2*	*e3-fs*	*e4-SORE1*	*dt1-tb*
Dongda 1	North-east China	*e1-as*	*e2*	*e3-fs*	*e4-kes*	***Dt1***
Heihe 12	North-east China	*e1-as*	*e2*	*e3-tr*	*e4-SORE1*	***Dt1***
Heihe 13	North-east China	*e1-as*	*e2*	*e3-fs*	*e4-kes*	***Dt1***
Heihe 21	North-east China	*e1-as*	*e2*	*e3-tr*	*e4-SORE1*	***Dt1***
Heihe 33	North-east China	*e1-as*	*e2*	*e3-tr*	*e4-kes*	***Dt1***
Heihe 35	North-east China	*e1-as*	*e2*	*e3-fs*	*e4-kes*	***Dt1***
Heihe 40	North-east China	*e1-as*	*e2*	*e3-fs*	*e4-kes*	***Dt1***
Heihe 41	North-east China	*e1-as*	*e2*	*e3-fs*	*e4-kes*	***Dt1***
Jiagedaqi 04	North-east China	*e1-as*	*e2*	*e3-fs*	*e4-SORE1*	***Dt1***
Jiagedaqi 05	North-east China	*e1-as*	*e2*	*e3-fs*	*e4-SORE1*	***Dt1***
Jiagedaqi 08	North-east China	*e1-as*	*e2*	*e3-tr*	*e4-SORE1*	***Dt1***
Jiagedaqi 11	North-east China	*e1-as*	*e2*	*e3-tr*	*e4-SORE1*	***Dt1***
Jiagedaqi 12	North-east China	*e1-as*	*e2*	*e3-tr*	*e4-SORE1*	***Dt1***
Jiagedaqi 14	North-east China	*e1-as*	*e2*	*e3-tr*	*e4-SORE1*	***Dt1***
Jiagedaqi 16	North-east China	*e1-as*	*e2*	*e3-tr*	*e4-kes*	***Dt1***
Jiagedaqi 17	North-east China	*e1-as*	*e2*	*e3-fs*	*e4-kes*	***Dt1***
Jiagedaqi 18	North-east China	*e1-as*	*e2*	*e3-fs*	*e4-kes*	***Dt1***
Jiagedaqi 19	North-east China	*e1-as*	*e2*	*e3-tr*	*e4-kes*	***Dt1***
Kiev 242 WH	Ukraine	*e1-as*	*e2*	*e3-tr*	*e4-SORE1*	*dt1-tb*
Nawiko	Poland	*e1-as*	*e2*	*e3-ns*	*e4-SORE1*	*dt1-tb*
Oktyabr-70	Far-east Russia	*e1-as*	*e2*	*e3-fs*	*e4-kes*	***Dt1***
Sadovy	Far-east Russia	*e1-as*	*e2*	*e3-fs*	*e4-kes*	***Dt1***
Vega	Far-east Russia	*e1-as*	*e2*	*e3-fs*	*e4-kes*	***Dt1***
Yug 30	Ukraine	*e1-as*	*e2*	*e3-tr*	*e4-SORE1*	***Dt1***
Zeya 2	Far-east Russia	*e1-as*	*e2*	*e3-tr*	*e4-kes*	***Dt1***
Heihe 34	North-east China	*e1-as*	*e2*	*e3-tr*	***E4***	***Dt1***
Jiagedaqi 02	North-east China	*e1-as*	*e2*	*e3-tr*	***E4***	***Dt1***
Jiagedaqi 20	North-east China	*e1-as*	*e2*	*e3-tr*	***E4***	***Dt1***
Dya-1	Far-east Russia	*e1-as*	*e2*	*e3-fs*	***E4***	***Dt1***
Sonata	Far-east Russia	*e1-as*	*e2*	*e3-fs*	***E4***	***Dt1***
Yubileinaya	Far-east Russia	*e1-as*	*e2*	*e3-fs*	***E4***	***Dt1***
Zeika	Far-east Russia	*e1-as*	*e2*	*e3-fs*	***E4***	***Dt1***
Gai	Poland	*e1-nl*	*e2*	*e3-tr*	*e4-SORE1*	***Dt1***
Karafuto 1	Hokkaido/Japan	*e1-nl*	*e2*	*e3-tr*	*e4-SORE1*	*dt1-bb*
Dongda 2	North-east China	*e1-nl*	*e2*	*e3-tr*	***E4***	***Dt1***
Heihe 28	North-east China	*e1-nl*	*e2*	*e3-tr*	***E4***	***Dt1***
Jiagedaqi 01	North-east China	*e1-nl*	*e2*	*e3-tr*	***E4***	***Dt1***
Jiagedaqi 03	North-east China	*e1-nl*	*e2*	*e3-tr*	***E4***	***Dt1***
Jiagedaqi 09	North-east China	*e1-nl*	*e2*	*e3-tr*	***E4***	***Dt1***
Jiagedaqi 10	North-east China	*e1-nl*	*e2*	*e3-tr*	***E4***	***Dt1***
Jiagedaqi 13	North-east China	*e1-nl*	*e2*	*e3-tr*	***E4***	***Dt1***
Sakamotowase	Hokkaido/Japan	*e1-fs*	*e2*	*e3-tr*	***E4***	*dt1-bb*
Kiev 242 BH	Ukraine	*e1-nl*	*e2*	***E3***	*e4-SORE1*	***Dt1***
Ustya	Ukraine	*e1-nl*	*e2*	***E3***	*e4-SORE1*	***Dt1***
