# Supplementary material for: Genetic variation in four maturity genes affects photoperiod insensitivity and PHYA-regulated post-flowering responses of soybean
Source: BMC Plant Biol. 2013 Jun 25;13:91. doi: 10.1186/1471-2229-13-91 (PMC3698206; doi:10.1186/1471-2229-13-91)
Supplement: Additional file 2 — Allele-specific DNA markers that distinguish recessive alleles from dominant functional ones at the maturity loci E1, E2, E3, and E4 and the determinate growth habit locus Dt1 in soybean. [file 1471-2229-13-91-S2.pdf]

Additional file 2. Allele-specific DNA markers that distingush resessive alleles from dominnant functional ones at the maturity loci, *E1* , *E2* , *E3* and *E4* , and a determinate growth habit locus, *Dt1,* in soybean

Locus	Allele		Primer sequence (5' - 3')	Marker type	Restriction enzyme	Sizes of amplified fragmengts and digested fragments (bp)		Reference
E1	*e1-fs* /*e1-nl*	F	CACTCAAATTAAGCCCTTTCA	CAPS	*Hinf* I	*E1/e1-as*	186 + 36	[13], this study
		R	TTCATCTCCTCTTCATTTTTGTTG			*e1-fs*	136 + 46 + 36	
						e1-nl	No amplification	
	e1-as	F	TCAGATGAAAGGGAGCAGTGTCAAAAGAAGT	dCAPS	*Taq* I	*E1/e1-fs* /*e1-nl*	444/443	[13]
		R	TCCGATCTCATCACCTTTCC			*e1-as*	413+31	
E2	*e2*	F	AAGCCTATGCCAGCTAGGTATTT	dCAPS	*Dra* I	*E2*	110	[29]
		R	GAAGCCCATCAGAGGCATGTCTTATT			*e2*	27+83	
E3	*e3-tr*	F	TGGAGGGTATTGGATGATGC	FLP		*E3-Misuzudaizu*	1,339	[30]
		R1	CTAAGTCCGCCTCTGGTTTCAG			*E3-Harosoy*	558	
		R2	CGGTCAAGAGCCAACATGAG			*e3-tr*	275	
		R3	GTCCTATACAATTCTTTACGACG					
	e3-fs	F	GGGATAGTTCTGATGCTGTTCAA	CAPS	*Ale* I	*E3*	552 + 206	This study
		R	CCTTGTATCGATAGCATATGTGCT			*e3-fs*	759	
	e3-ns	F	GTTGAAGAGAAGATCACAACA	dCAPS	*Mfe* I	*E3*	163	This study
		R	GATGAACTAATTTCCCTAACTGCA			*e3-ns*	140 + 23	
E4	*e4-oto*	F	CCCAGACACTCTTGTGTGAT	CAPS	*Sac* I	*E4*	535	[31]
		R	CCATACTCTCGGTATCTTTG			*e4-oto*	439 + 96	
	e4-tsu	F	CACCCTAGGAGTTGTGTTGTT	dCAPS	*Eco* RV	*E4*	355	[31]
		R	GCGGTTCTGTACAATTGCCTGATA			*e4-tsu*	332 + 23	
	e4-kam	F	CTTAATAAAGCCATGACTGGTTTG	CAPS	*Afl* II	*E4*	494	[31]
		R	CTTGAGTTTCAATGAGGTTTCAAC			*e4-Kam*	286 + 208	
	e4-kes	F	CTTAATAAAGCCATGACTGGTTTG	CAPS	*Bsp* HI	*E4*	494	[31]
		R	CTTGAGTTTCAATGAGGTTTCAAC			*e4-Kes*	399 + 95	
	e4-SORE-1	F	AGACGTAGTGCTAGGGCTAT	FLP		*E4*	1,229	[7]
		R1	GCATCTCGCATCACCAGATCA			*e4-SORE-1*	837	
		R2	GCTCATCCCTTCGAATTCAG					
Dt1	*dt1-ab*	F	CACACCCACCCCACATATAT	CAPS	*Hind* III	*Dt1*	808	This study
		R	GGCAAAACCAGCAGCTACTT			*dt1-ab*	593 + 215	
	dt1-tb	F	CACACCCACCCCACATATAT	CAPS	*Acc* I	*Dt1*	646 + 92 + 70	This study
		R	GGCAAAACCAGCAGCTACTT			*dt1-tb*	738 + 70	
	dt1-bb	F	GGCTGCTGTCTACTTCAATGTCTAG	dCAPS	*Xba* I	*Dt1*	155+21	[33]
		R	GCCACATGTGAAGATCAACTTCCA			*dt1-bb*	176	

CAPS; cleaved amplified polymorphic sequence, FLP; fragment length polymorphism
